# Subclinical thyroid dysfunction and incident diabetes: a systematic review and an individual participant data analysis of prospective cohort studies

**DOI:** 10.1530/EJE-22-0523

**Published:** 2022-09-30

**Authors:** Heba Alwan, Fanny Villoz, Martin Feller, Robin P F Dullaart, Stephan J L Bakker, Robin P Peeters, Maryam Kavousi, Douglas C. Bauer, Anne R Cappola, Bu B Yeap, John P Walsh, Suzanne J Brown, Graziano Ceresini, Luigi Ferrucci, Jacobijn Gussekloo, Stella Trompet, Massimo Iacoviello, Jae Hoon Moon, Salman Razvi, Isabela M. Bensenor, Fereidoun Azizi, Atieh Amouzegar, Sergio Valdés, Natalia Colomo, Nick J Wareham, J Wouter Jukema, Rudi G J Westendorp, Ki Woong Kim, Nicolas Rodondi, Cinzia Del Giovane, for the Thyroid Studies Collaboration

**Affiliations:** 1 Institute of Primary Health Care (BIHAM), University of Bern, Bern, Switzerland; 2 Graduate School for Health Sciences, University of Bern, Mittelstrasse 43, 3012, Bern, Switzerland; 3 Department of Internal Medicine, University Medical Center Groningen, University of Groningen, Groningen, The Netherlands; 4 Department of Internal Medicine, Erasmus MC, Rotterdam, the Netherlands; 5 Department of Epidemiology, Erasmus MC, University Medical Center Rotterdam, Rotterdam, the Netherlands; 6 Departments of Medicine and Epidemiology & Biostatistics, University of California, San Francisco, California, United States; 7 Division of Endocrinology, Diabetes, and Metabolism, Department of Medicine, University of Pennsylvania School of Medicine, Philadelphia, Pennsylvania, United States; 8 Medical School, University of Western Australia, Perth, Australia; 9 Department of Endocrinology and Diabetes, Fiona Stanley Hospital, Perth, Australia; 10 Discipline of Internal Medicine, Medical School, University of Western Australia, Perth, Australia; 11 Department of Endocrinology and Diabetes, Sir Charles Gairdner Hospital, Perth, Western Australia, Australia; 12 Department of Medicine and Surgery, University of Parma, Italy; 13 National Institute on Aging, National Institutes of Health, Baltimore, Maryland, United States; 14 Section Gerontology and Geriatrics, Department of Internal Medicine, Leiden University Medical Center, Leiden, the Netherlands; 15 Department of Public Health and Primary Care, Leiden University Medical Center, Leiden, the Netherlands; 16 Department of Medical and Surgical Sciences, University of Foggia, Foggia, Italy; 17 Department of Internal Medicine, Seoul National University Bundang Hospital, Seoul National University College of Medicine, Soeul, South Korea; 18 Translational and Clinical Research Institute, Newcastle University, Newcastle upon Tyne, United Kingdom; 19 Center for Clinical and Epidemiologic Research, University Hospital of São Paulo, São Paulo, Brazil; 20 Endocrine Research Center, Research Institute for Endocrine Sciences, Shahid Beheshti University of Medical Sciences, Tehran, Iran; 21 Department of Endocrinology and Nutrition, Hospital Regional Universitario de Málaga/Universidad de Málaga, Instituto de Investigación Biomedica de Málaga-IBIMA, Málaga, Spain; 22 CIBERDEM, Instituto de Salud Carlos III Spain,; 23 MRC Epidemiology Unit, Institute of Metabolic Sciences, University of Cambridge, Cambridge, UK; 24 Department of Cardiology, Leiden University Medical Center, Leiden, The Netherlands; 25 Netherlands Heart Institute, Utrecht, the Netherlands; 26 Department of Public Health and Center of Healthy Ageing, University of Copenhagen, Denmark; 27 Department of Neuropsychiatry, Seoul National University Bundang Hospital, Seongnam, South Korea; 28 Department of Brain and Cognitive Science, Seoul National University College of Natural Sciences, Seoul, South Korea; 29 Department of Psychiatry, Seoul National University, College of Medicine, Seoul, South Korea; 30 Department of General Internal Medicine, Inselspital, Bern University Hospital, University of Bern, Bern, Switzerland

**Keywords:** diabetes, incidence, subclinical thyroid dysfunction, thyroid hormones

## Abstract

**Objective:**

Few prospective studies have assessed whether persons with subclinical thyroid dysfunction are more likely to develop diabetes, with conflicting results. We conducted a systematic review of the literature and an individual participant data analysis of multiple prospective cohorts to investigate the association between subclinical thyroid dysfunction and incident diabetes.

**Methods:**

We performed a systematic review of the literature in Medline, Embase, and the Cochrane Library from inception to February 11 2022. A two-stage individual participant data analysis was conducted to compare participants with subclinical hypothyroidism and subclinical hyperthyroidism versus euthyroidism at baseline and the adjusted risk of developing diabetes at follow-up.

**Results:**

Among 61,178 adults from 18 studies, mean age was 58 years, 49% were females, and mean follow-up time was 8.2 years. At last available follow-up, there was no association between subclinical hypothyroidism and incidence of diabetes (OR=1.02, 95% confidence interval (CI): 0.88-1.17, I^2^=0%), or subclinical hyperthyroidism and incidence of diabetes (OR=1.03, 95% CI: 0.82-1.30, I^2^=0%), in age- and sex-adjusted analyses. Time-to-event analysis showed similar results (hazard ratio for subclinical hypothyroidism: 0.98, 95% CI: 0.87-1.11; hazard ratio for subclinical hyperthyroidism: 1.07, 95% CI: 0.88-1.29). The results were robust in all subgroup and sensitivity analyses.

**Conclusions:**

This is the largest systematic review and individual participant data analysis to date investigating the prospective association between subclinical thyroid dysfunction and diabetes. We did not find an association between subclinical thyroid dysfunction and incident diabetes. Our results do not support screening patients with subclinical thyroid dysfunction for diabetes.

**Registration:**

Prospero CRD 42021259695

## Introduction

Thyroid dysfunction and diabetes are two of the most common endocrine diseases and studies have suggested that these two disorders tend to co-exist more frequently than expected by chance (1). Subclinical hypothyroidism (Shypo) is defined as an elevated serum thyroid stimulating hormone (TSH) with serum free thyroxine (fT4) concentrations within the reference range (2). Shypo is a common disorder that affects up to 10% of the adult population (2) and has been associated with an increased risk of cardiovascular disease events and mortality (3). On the other hand, subclinical hyperthyroidism (Shyper) is diagnosed when serum TSH is low with fT4 and free triiodothyronine (fT3) concentrations in the reference range (2) and has also been associated with adverse events (4).

Results from cross-sectional studies on the association between diabetes and thyroid disease have been conflicting. A large cross-sectional study conducted in Norway among more than 30,000 individuals did not reveal an association between hypothyroidism and type 2 diabetes (5). Conversely, other cross-sectional studies have found an association between raised serum TSH levels and insulin resistance (6, 7). However, cross-sectional studies have several limitations including potential confounding by reverse causation. Moreover, it has been suggested that diabetes and thyroid disease have a bidirectional relationship (8, 9). Only a few longitudinal studies have investigated the association between thyroid dysfunction and incident diabetes with, again, conflicting results, and most studies only included individuals with overt thyroid disease. One prospective study conducted in the Netherlands found that higher TSH levels were associated with a higher risk of developing diabetes, particularly among individuals with pre-diabetes (10). Two other longitudinal studies did not find an association between Shypo and incidence of metabolic syndrome (11, 12). A recent meta-analysis of prospective studies found that there was no association between thyroid function and risk of type 2 diabetes when TSH was analyzed as a continuous variable (13). This study, however, did not specifically analyze SCTD as a predictor of diabetes.

The conflicting results from the literature may be explained by lack of power among studies as well as differences in definitions of exposure and outcome and statistical methods. Individual participant data (IPD) analysis allows researchers to standardize definitions and methods across studies, as well as conduct subgroup analyses while also increasing statistical power (14). We therefore conducted a systematic review of the literature and an IPD analysis to explore whether individuals with SCTD are more prone to develop diabetes as compared to euthyroid individuals using data from prospective international cohort studies.

## Materials and methods

This systematic review and IPD analysis was registered in the international Prospective Register of Systematic Reviews PROSPERO (CRD 42021259695). We adhered to the Preferred Reporting Items for Systematic reviews and Meta-Analyses (PRISMA) statement for IPD systematic reviews (15).

### Search strategy and selection criteria

We performed a systematic literature search in Ovid Medline, Ovid Embase, and in the Cochrane Library from inception to February 11, 2022. We included publications from prospective studies that had data on baseline TSH in adults and that assessed incidence of diabetes during follow-up. The search strategy combined terms related to exposure (e.g. thyroid diseases, hyperthyroidism, hypothyroidism, thyroid hormones, triiodothyronine, thyroxine, thyrotropin, subclinical, mild) and outcome (e.g. diabetes, metabolic syndrome, insulin resistance, pre-diabetes). Details of the search strategy are presented in the Appendix. We excluded: (1) studies that only included participants with normal thyroid function at baseline, (2) studies that only included participants with overt thyroid dysfunction at baseline, (3) studies without a euthyroid control group, (4) studies that only included participants who took thyroid-altering medications, (5) and studies that included only participants less than 18 years old or pregnant women. We only included studies published in English. Two authors (H.A. and F.V.) screened all references for eligibility and discrepancies were resolved by consensus with a third author (C.D.G.). Additional unpublished data were also identified from the Thyroid Studies Collaboration (TSC), a consortium of cohort studies that study the association between SCTD and various clinical outcomes (3).

### Data extraction and quality assessment

Studies that met the inclusion criteria were invited to collaborate in the present IPD analysis by sharing their data. We requested data on thyroid function at baseline (TSH, fT4, and when available, fT3), demographics, anthropometrics, medication use (levothyroxine, anti-thyroid medication, thyroid-altering medication, anti-diabetic medication), cardiovascular risk factors, and biochemical data to define diabetes. Thyroid medication was defined as levothyroxine or anti-thyroid medication use, and thyroid-altering medication was defined as levothyroxine, anti-thyroid medication, lithium, or amiodarone use. Each study was approved by its local ethics committee (Supplementary Table 1). The Newcastle-Ottawa Scale (NOS) was used to assess the quality of the included studies (16). The NOS contains eight items divided into three categories: Selection, Comparability, and Outcome. Studies are given a score ranging from 0 to 9 stars with the highest score indicating the best methodological quality. Studies were classified into good, fair, and poor quality according to their star rating. The GRADE tool was also used to assess the certainty in the evidence (www.gradeworkinggroup.org) (17). To assess the Study Limitations (Risk of Bias) domain in the GRADE, we used the final NOS score. For example, if a study had a good NOS score, the Study Limitations domain in the GRADE would be considered as “not serious”. Publication bias was explored with funnel plots and Egger’s test.

### Exposures

The exposures in this study were Shypo and Shyper as compared to euthyroidism. As previously done in IPD analyses from the TSC (3, 18), we used uniform TSH cut-off levels and study-specific fT4 cut-off values to define thyroid status as fT4 assays show greater inter-method variation than 3^rd^ generation TSH assays. Euthyroidism was defined as a TSH from 0.45 to 4.49 mIU/L, subclinical hyperthyroidism as a TSH <0.45 mlU/L with fT4 in the reference range and subclinical hypothyroidism as TSH > 4.5 mlU/L with fT4 in the reference range. Participants with fT4 values out of the reference range were excluded from the analyses. Participants with missing fT4 values but with TSH levels below 0.45 mIU/l were considered to have subclinical rather than overt hyperthyroidism and participants who had missing fT4 values but TSH levels between 4.5 mIU/L and 19.9 mIU/L were considered to have subclinical rather than overt hypothyroidism. This strategy was adopted as individuals with TSH in these ranges are most likely to have subclinical rather than overt thyroid dysfunction (19, 20). We also used study-defined cut-offs to define positivity of thyroid peroxidase antibodies (TPOAb).

### Outcomes

Our primary outcome was incident diabetes at the last available follow-up. Diabetes was defined according to the American Diabetic Association criteria as either: (i) fasting plasma glucose ≥ 7 mmol/l, (ii) 2-hour glucose ≥ 11.1 mmol/L after an oral glucose tolerance test (OGTT), (iii) glycated hemoglobin (HbA1c) ≥ 6.5% (48 mmol/mol) (21), or use of blood glucose lowering medication. Self-reported diabetes cases without ascertainment by biochemical data or medication use were not included in the primary analysis. Although data on the type of diabetes (1 versus 2) was not available, we considered that most incident diabetes cases were type 2 diabetes. In the analysis of incident diabetes, we excluded participants with diabetes at baseline. We also excluded participants with missing data on thyroid status at baseline and diabetes status at baseline and follow-up. Secondary outcomes included incident diabetes at first available follow-up; incidence of pre-diabetes at first and last available follow-ups and time to diabetes. Pre-diabetes was defined according to the American Diabetic Association criteria as either: (i) fasting plasma glucose ≥ 5.6 mmol/l, (ii) 2-hour glucose ≥ 7.8 mmol/L after an OGTT, or a (iii) HbA1c ≥ 5.7% (39 mmol/mol) without meeting criteria for diabetes (21). Time to event of newly developed diabetes was measured from baseline TSH measurement to the date of the study visit when diabetes was ascertained (using biochemical data or self-report of anti-diabetic medication use), or when available, date of diagnosis of diabetes.

### Statistical analysis

We conducted a two-stage IPD analysis. In the first stage, the effect size for each cohort was estimated, and in the second stage, they were pooled together using a random effects model. For the primary outcome, we assessed the association between Shypo and Shyper and incident diabetes at last available follow-up by calculating the odds ratio (OR) using a logistic regression model adjusted for age and sex. In line with previous studies investigating the association between thyroid function and diabetes (10, 22), we ran a multivariable model adjusting further for smoking, blood pressure, total cholesterol, body mass index (BMI), and baseline fasting blood glucose as a secondary analysis. For the time to event outcome, we used a Coxproportional hazards model and results were presented as hazard ratios (HR) as compared to the reference category (euthyroid individuals). Finally, for one cohort where IPD was not available (22), aggregate data was added in the second stage of the IPD analysis to assess the association between Shypo and incident diabetes (data was not available for Shyper).

We also conducted pre-defined sub-group analyses on the primary outcome to identify possible sources of heterogeneity. We performed subgroup analyses by age (younger and older than 65 years), by sex, and by TSH levels (for Shypo: 4.50–6.99 mIU/L, 7.00–9.99 mIU/L, 10.0–19.9 mIU/L, and for Shyper: 0.1-0.45 mIU/l, <0.1 mIU/l). We also stratified participants by thyroid peroxidase antibody status (TPOAb; positive versus negative). The latter sub-group analysis was not described in the PROSPERO protocol.

The following sensitivity analyses were performed: excluding participants with thyroidaltering drugs or thyroid-hormone replacement at baseline; requiring fT3 (available in 6 cohorts) as well as fT4 to be within range to define Shyper; and limiting analyses to high-quality studies (i.e. studies that were classified as good quality using the NOS). The following sensitivity analyses were not originally described in the PROSPERO protocol but were subsequently added: limiting analyses to participants who have persistent Shypo and Shyper at follow-up, limiting analyses to studies with less than 20% missing data at follow-up, and for studies where additional data was available on diabetes status (i.e. self-reported diabetes or diabetes ascertainment using medical records), the definition of diabetes was extended to include this information as a sensitivity analysis.

We estimated heterogeneity using I^2^ and the Q test. A P-value <0.05 was considered statistically significant. Stata 16.0 (StataCorp LP, College Station, TX) was used to conduct all analyses.

## Results

Of the 2334 studies identified through the literature search, four studies met our inclusion criteria (10–12, 22) (Supplementary Figure 1). We further identified 15 additional studies from other sources including from within the TSC (6, 23–36). We then invited the principal investigators of the identified studies (n=19) to be included in the present IPD analysis. All but one study (22) which were identified through the literature search and from within the TSC accepted to participate. We received IPD from 18 studies from Europe, North America, Australia, and Asia. Study characteristics and baseline data of the 18 studies included in the IPD analysis are displayed in Table 1. After excluding individuals with confirmed diabetes at baseline, missing thyroid function or diabetes data at baseline, and individuals with overt hypothyroidism or hyperthyroidism at baseline, 61,178 participants were included in the analyses. Mean age was 58 years (range 18-105) and 49% were women. Mean BMI was 26 kg/m^2^ (range 13-59). At baseline, 90% of participants were euthyroid, 7% of participants had Shypo, and 3% of participants had Shyper. Out of 39,742 individuals with available data at the last available follow-up (mean duration of 8.2 years), 2,910 individuals (7.3%) developed diabetes. As none of the participants in the Shypo and Shyper groups developed diabetes in the Bari study, we were unable to include data from this study in further analyses.

### Subclinical hypothyroidism

**Figure 1:**
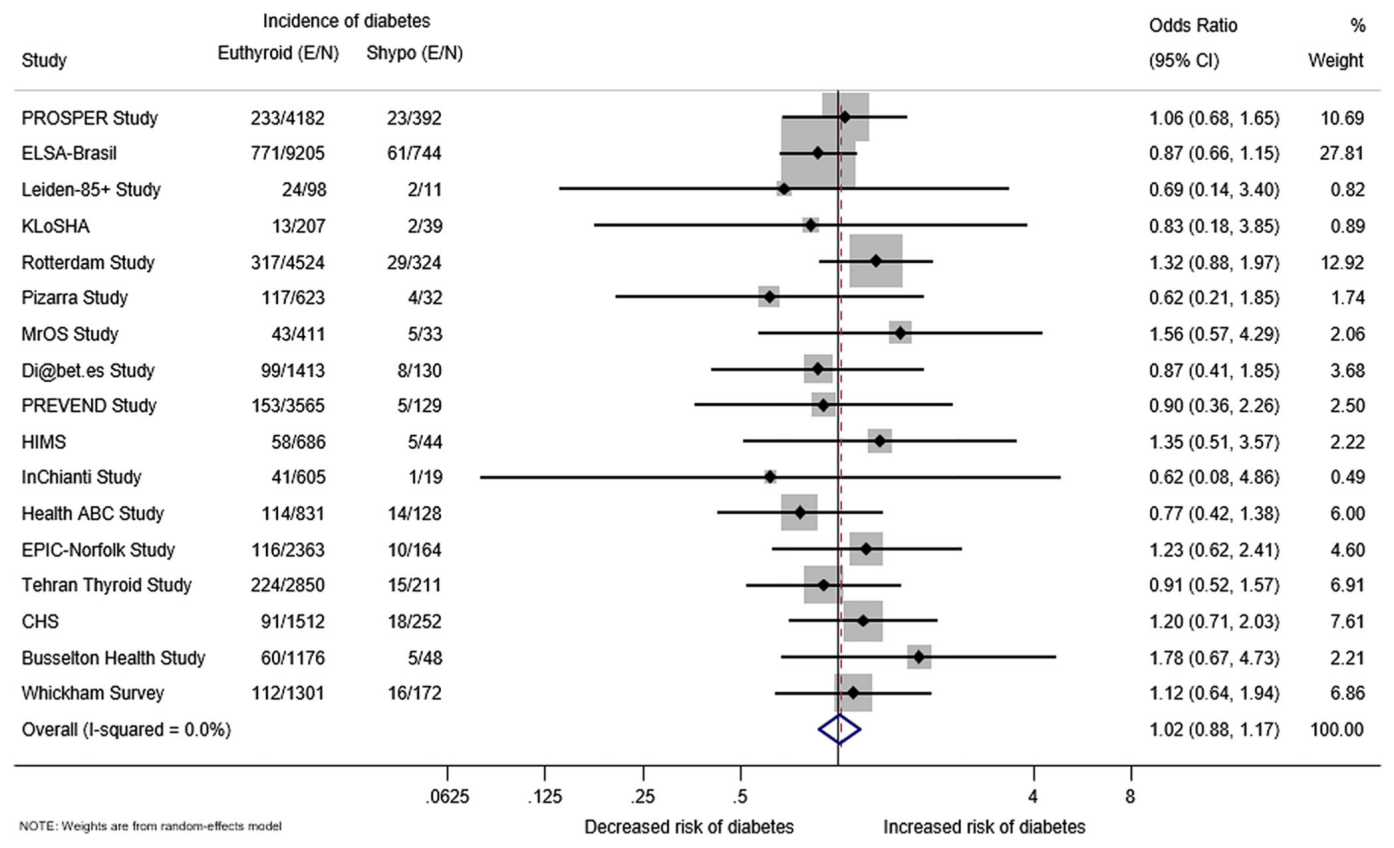
Age and sex- adjusted logistic regression analysis of individual participant data of the association between subclinical hypothyroidism and incident diabetes at the last available follow-up E/N: Number of events/Total number of participants at last available follow-up; Shypo: subclinical hypothyroidism; Cl: confidence interval; PROSPER: Prospective Study of Pravastatin in the Elderly at Risk Study; ELSA-Brasil: Brazilian Longitudinal Study of Adult Health; Leiden 85+ Study: Leiden 85-plus Study; PREVEND: Prevention of Renal and Vascular End-stage Disease Study; HIMS: Health in Men Study; InChianti: Invecchiare in Chianti Study; Health ABC Study: The Health, Aging and Body Composition Study; EPIC-Norfolk Study: European Prospective Investigation into Cancer - Norfolk Study; CHS: Cardiovascular Health Study 201×156mm (120 × 120 DPI)

IPD age- and sex-adjusted analysis for the association between Shypo at baseline and incident diabetes at last available follow-up among 17 cohorts (N=36,424) is shown in Figure 1. We found no association between Shypo at baseline and incident diabetes (OR = 1.02; 95% confidence interval (CI): 0.88-1.17). The I^2^ statistic was 0%, indicating low heterogeneity among studies.

The associations between Shypo and various secondary outcomes are displayed in Table 2. There was no association between Shypo and incidence of pre-diabetes at last available follow-up (OR = 0.94; 95% CI: 0.84-1.05). Similarly, no association was found between Shypo and diabetes or pre-diabetes at first available follow-up (OR = 1.02; 95% CI: 0.88-1.17 for diabetes and OR = 0.96; 95%I CI: 0.85-1.09 for prediabetes). In the Cox regression model, the HR for developing diabetes was 0.98 (95%CI: 0.87-1.11). Multivariable analysis adjusted for age, sex, systolic and diastolic blood pressure, fasting blood glucose (or if not available, HbA1c or OGTT), smoking, total cholesterol, and BMI showed similar results to age-and sex-adjusted analyses for the association between Shypo and diabetes incidence at last available follow-up (OR = 0.97; 95%CI: 0.82-1.13). Including aggregate data from 54,333 euthyroid or Shypo participants of the study by Gronich et al (22) (total N = 90,757) did not change the results.

Sensitivity analyses for Shypo are shown in Table 3. After excluding participants taking thyroid medication or missing thyroid medication data (N=23,992 after exclusion) or thyroid-altering medication or missing thyroid-altering medication data (N=16,971 after exclusion) at baseline and limiting the analyses to participants who had repeat thyroid function testing to confirm the persistence of Shypo at follow-up (N=17,441) revealed similar results to our primary analysis. Moreover, results from a sensitivity analysis where additional data when available (from medical records or selfreported diabetes status) was used to define incident diabetes (N=55,652) again revealed no association between Shypo and incident diabetes. Finally, limiting analyses to studies with less than 20% missing data at follow-up did not change our results.

**Figure 3:**
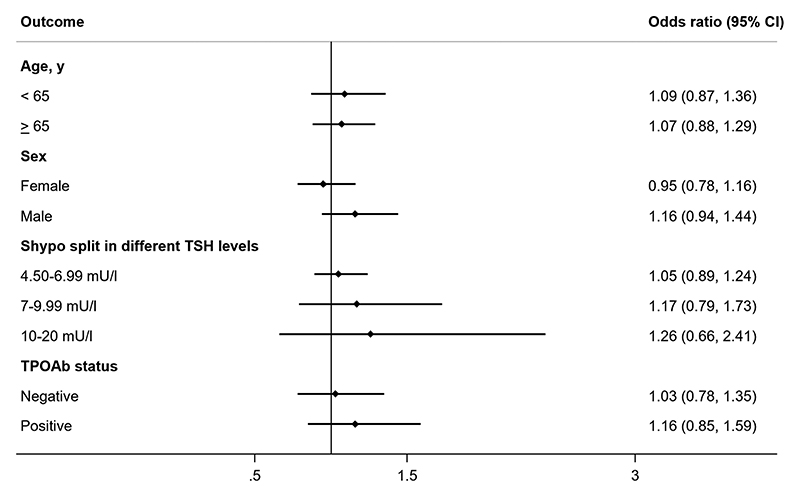
Association between subclinical hypothyroidism and incident diabetes at last available follow-up by subgroups 173×126mm (300 × 300 DPI)

Several subgroup analyses for Shypo are displayed in Figure 3. Stratifying participants according to age (below and above 65 years of age), sex, TSH levels, and TPOAb status did not show different results as compared to the primary analysis.

### Subclinical hyperthyroidism

**Figure 4:**
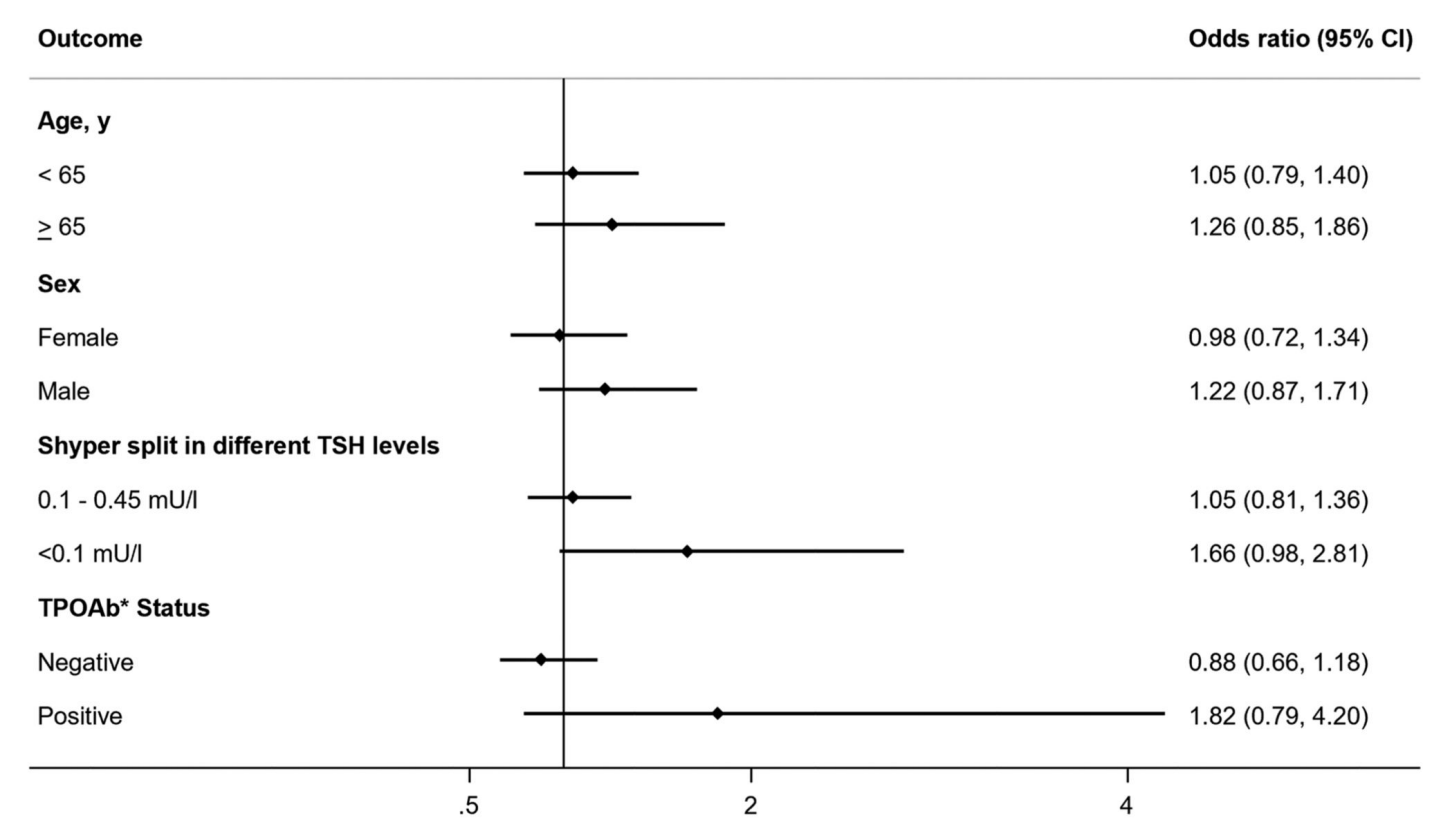
Association between subclinical hyperthyroidism and incident diabetes at last available follow-up by subgroups 171×124mm (300 × 300 DPI)

Data from 12 cohorts (N=32,747) showed the age- and sex-adjusted OR for the association between having Shyper at baseline and developing diabetes at last available follow-up was 1.03 (95% CI: 0.82-1.30, I^2^ = 0%) (Figure 1). There was also no significant association between Shyper and incident diabetes at first available follow-up (OR = 1.07; 95%I CI: 0.82-1.40), or pre-diabetes at last and first available follow-up (OR = 1.03; 95% CI: 0.85-1.25 and OR = 1.03 (95% CI: 0.89-1.19, respectively) (Table 2). The HR for incidence of diabetes at last available follow-up for individuals with Shyper was 1.07 (95% CI: 0.88-1.29). The results were similar for several sensitivity and sub-group analyses (Table 4, Figure 4).

### Quality assessment

The quality of all studies included in the analyses was good according to the NOS (Supplementary Table 2). Based on the GRADE tool, certainty in the evidence for the primary outcome was low due to the observational nature of all studies (Supplementary Table 3). Funnel plots and Egger’s test for the primary outcome did not suggest the presence of publication bias or a small study effect (Supplementary Figures 2 and 3).

## Discussion

In this large IPD analysis of 61,178 participants, we did not find a prospective association between SCTD at baseline and incident diabetes or pre-diabetes at followup. Our results remained consistent in all sub-group and sensitivity analyses. To our knowledge, this is the first IPD to date investigating the association between SCTD and incident diabetes.

Our results are consistent with findings from a study conducted in Iran which did not find an association between TSH and fT4 in the subclinical thyroid range and fasting blood glucose during follow-up (11). In line with our findings, a large prospective study conducted in Taiwan found that high TSH was not associated with incidence of diabetes (37). However, unlike the results from our study, the authors found that high TSH was associated with incidence of pre-diabetes, although analyses were not restricted to individuals with subclinical thyroid dysfunction (37). Results from the Rotterdam study were in contrast to our findings as they showed that higher TSH was associated with an increased risk of diabetes (HR = 1.13; 95% CI: 1.08–1.18 per 1 SD increase in log TSH) (10). It is noteworthy however that the authors also included TSH within the reference range and overt thyroid disease which may explain the difference in results. Interestingly, a registry-based study in Israel found that Shypo was associated with incident diabetes only among statin users, and not among statin nonusers (22). The authors suggested that both Shypo and diabetes can be associated with mitochondrial dysfunction, which can be worsened by statin use (22). It has been postulated that diabetes and thyroid dysfunction may have a bidirectional effect on each other (8, 9, 13). In theory, there are multiple underlying mechanisms that can explain how SCTD can contribute to the development of diabetes. First, hypothyroidism, both overt and subclinical, is associated with increased insulin resistance in part due to a decreased glucose uptake in muscle and adipose tissue (1). Moreover, TSH stimulates hepatic glucose production and reduces insulin secretion from pancreatic beta cells which in turns leads to higher serum glucose levels (1). Conversely, hyperglycemia can have an effect on thyroid hormones by controlling TSH secretion from the hypothalamus, influencing conversion of fT4 to fT3 in peripheral tissues, and affecting the TSH response to thyrotropin-releasing hormone (TRH) (1). Moreover, it has been shown that raised serum insulin levels can lead to an increase in thyroid volume (9). In line with these mechanisms, a meta-analysis of cross-sectional studies by Han et al in 2015 showed that diabetes was associated with a 1.93-fold increase in risk of Shypo (38). Moreover, a study conducted in Australia among 420 women with diabetes found that 8.6% had Shypo (39). It is therefore possible that the diabetic state may contribute to the development of SCTD, which can explain the cross-sectional association between Shypo and diabetes that has been reported in the literature. Longitudinal studies assessing the prospective association between the presence of diabetes at baseline and SCTD at follow-up are thus warranted.

Our results have clinical implications as they do not support screening patients with SCTD for diabetes nor treating them in the hope of preventing diabetes in the future. This can therefore avoid performing unnecessary tests on patients and overtreating them which can lead to unwanted side-effects.

Our study has several strengths, namely, it includes a large number of participants with a long mean follow-up time. As this study is an IPD analysis, we were able to standardize the definitions of SCTD and diabetes across studies and uniformly adjust for confounders to reduce heterogeneity across studies. We were also able to perform several sub-group analyses due to the large nature of this IPD. Moreover, we included unpublished data which increased the power of our study. However, our study also has limitations. Some studies included in our analysis were not designed to investigate the incident diabetes and therefore diabetes-related data were not collected for all participants at follow-up, which increased missing data during follow-up. However, we conducted a sensitivity analysis including only studies with less than 20% missing data during follow-up which showed that our results were robust. Moreover, SCTD was defined at a single time point (baseline) for the primary outcome. It is thus possible that some individuals only present SCTD for a limited time period and then revert back to normal thyroid function, or may progress to overt thyroid disease. However, a sensitivity analysis that included only participants that have persistent SCTD at followup demonstrated that our results remained unchanged.

In conclusion, in this large IPD analysis, we did not find an association between and incident diabetes. Based on these findings, screening patients with Shypo for diabetes or treating them with levothyroxine with the aim of preventing diabetes would not be indicated.

## Supplementary Material

Supplementary Material

## Figures and Tables

**Figure 2:**
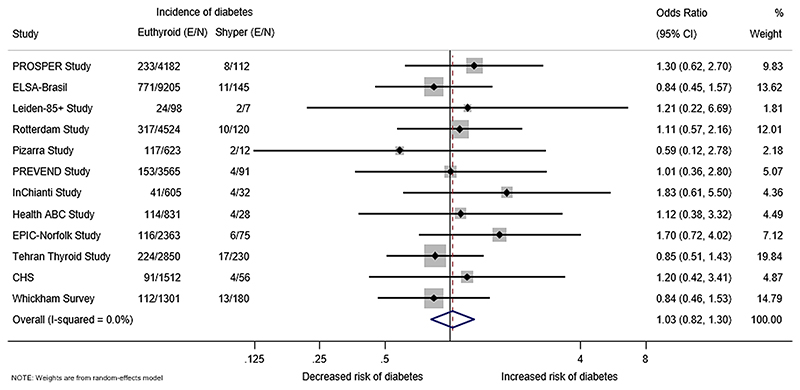
Age and sex- adjusted logistic regression analysis of individual participant data of the association between subclinical hyperthyroidism and incident diabetes at the last available follow-up E/N: Number of events/Total number of participants at last available follow-up; Shyper: subclinical hyperthyroidism; Cl: confidence interval; PROSPER: Prospective Study of Pravastatin in the Elderiy at Risk Study; ELSA-Brasil: Brazilian Longitudinal Study of Adult Health; Leiden 85+ Study: Leiden 85-plus Study; PREVEND: Prevention of Renal and Vascular End-stage Disease Study; HIMS: Health in Men Study; InChianti: Invecchiare in Chianti Study; Health ABC Study: The Health, Aging and Body Composition Study; EPIC-Norfolk Study: European Prospective Investigation into Cancer - Norfolk Study; CHS: Cardiovascular Health Study 200×133mm (120 × 120 DPI)

**Table 1:** Study characteristics at baseline measurement of thyroid function

Study, Place	Participants, *n*	Age, mean (range), y	Women, *n* (%)	BMI, mean (range), kg/m2	Thyroid medication^†^, *n* (%)	Median TSH, mIU/l	Normal range FT4, pmol/l	Positive TPOAb, *n* (%)	Last available follow-up mean ±S.D., y
PROSPER Study, the Netherlands	4774	75 (69-83)	2316 (49)	27 (15-50)	159 (3)	1.9	12-18	N.A.	3.2 **±** 0.7
ELSA-Brasil, Brazil	10839	51 (35-74)	6004 (55)	27 (15-58)	675 (6)	2.0	12-22	1198 (11)	3.8**±** 0.4
BARI Study, Italy	170	61 (26-87)	37 (22)	28 (17-48)	24 (14)	2.2	9-23.2	N.A.	1.0**±** 0
Leiden-85+ Study, the Netherlands	232	85 (85-85)	70 (30)	27 (15-40)	11 (5)	1.6	13-23	N.A.	5.0**±** 0
KLoSHA Study, South Korea	560	77 (65-105)	309 (55)	23 (15-33)	5 (0.9)	2.6	11-23	31 (5)	5.0**±** 0
Rotterdam Study, the Netherlands	8251	64 (45-101)	4735 (57)	27 (13-54)	239 (3)	1.9	11-25	1054 (13)	8.3**±** 2.6
Pizarra Study, Spain	853	39 (18-66)	525 (62)	27 (16-47)	3 (0.4)	1.8	11-22	33 (4)	6.7**±** 1.5
MrOS, United States	1237	74 (65-99)	0 (0)	27 (17-45)	91 (8)	2.0	9-24	N.A.	6.9**±** 0.4
Di@bet.es Study, Spain	3827	49 (18-93)	2246 (59)	28 (14-61)	152 (4)	2.1	11-22	320 (8)	7.6**±** 0.5
PREVEND Study, the Netherlands	5692	52 (32-80)	2864 (50)	26 (17-58)	123 (3)	1.6	12-22	569 (10)	7.7**±** 0.8
HIMS, Australia	740	75 (70-87)	0 (0)	26 (15-40)	18 (2)	2.0	10-23	N.A.	8.7**±** 0.9
InChianti Study, Italy	1044	68 (21-102)	590 (57)	27 (18-43)	18 (2)	1.4	10-27	N.A.	9.0**±** 0.2
Health ABC Study, United States	2178	75 (69-81)	1138 (52)	27 (15-51)	218 (10)	2.1	10-23	N.A.	9.2**±** 0.1
EPIC-Norfolk Study, United Kingdom	8386	58 (40-78)	4596 (55)	26 (15-59)	5 (0.1)	1.7	9-20	N.A.	9.2**±** 0.9
Tehran Thyroid Study, Iran	4586	39 (20-86)	1895 (41)	26 (15-52)	81 (2)	1.6	12-20	527 (11)	9.8**±** 0.9
CHS, United States	3194	75 (64-98)	1921 (60)	26 (14-59)	250 (8)	2.2	9-22	413 (13)	6.0**±** 0
Busselton Health Study, Australia	1966	49 (18-90)	961 (49)	25 (16-45)	15 (0.8)	1.5	9-23	223 (11)	13**±** 0
Whickham Survey, England	2649	47 (18-93)	1391 (53)	25 (15-45)	101 (4)	2.1	3.6-13.6^*^	179 (7)^**^	21.9**±** 9.9
**Total**	**61178**	**58 (18-105)**	**29870 (49)**	**26 (13-59)**	**2188 (3)**	**1.8**	**N.A.**	**4547 (10.7)**	**8.2± 3.5**

PROSPER: prospective study of Pravastatin in the elderly at risk Study; ELSA-Brasil: Brazilian Longitudinal Study of Adult Health; Leiden 85+ Study: Leiden 85-plus Study; KLoSHA: Korean Longitudinal Study on Health and Aging Study; MrOS: Osteoporotic Fractures in Men Study; PREVEND: Prevention of Renal and Vascular End-stage Disease Study; HIMS: Health in Men Study; InChianti: Invecchiare in Chianti Study; Health ABC Study: The Health, Aging and Body Composition Study; EPIC-Norfolk Study: European Prospective Investigation into Cancer - Norfolk Study; CHS: Cardiovascular Health Study; TPOAb: thyroid peroxidase antibodies

* Total T4 (μg/dL);

** Antimicrosomal antibodies were used for the Whickham Survey as data on thyroid peroxidase antibodies was not available;

† Thyroid medication was defined as levothyroxine or anti-thyroid medication use

**Table 2:** Association between subclinical hypo- and hyperthyroidism and secondary outcomes

Secondary outcome/analysis	Subclinical hypothyroidism			Subclinical hyperthyroidism		
	Euthyroid (E/N)	Shypo (E/N)	OR (95% CI)	Euthyroid (E/N)	Shyper (E/N)	OR (95% CI)
Pre-diabetes at last available follow-up	6539/22423	527/1782	0.94 (0.84-1.05)	6534/22365	229/825	1.03 (0.85-1.25)
Diabetes at first available follow-up	2097/36485	166/2931	1.02 (0.88-1.17)	1869/33240	64/1182	1.07 (0.82-1.40)
Pre-diabetes at first available follow-up	4358/22707	374/1774	0.96 (0.85-1.09)	4344/23085	143/864	1.03 (0.89-1.19)
Multivariable analysis^*^	2586/33552	223/2872	0.97 (0.82-1.13)	2313/31659	85/1088	1.00 (0.78-1.28)
Incident diabetes including aggregate data from Gronich et al^22^	-/87693	-/3064	1.12 (0.94-1.33)†	-	-	-
Time to diabetes (Cox regression)	3240/42562	283/3464	0.98 (0.87-1.11)‡	2959/38572	117/1267	1.07 (0.88-1.29)‡

E/N: Number of events/Total number of participants at follow-up; Shypo: subclinical hypothyroidism; Shyper: subclinical hyperthyroidism; OR: odds ratio; CI: confidence interval; HR: hazard ratio

* Adjusted for age, sex, systolic blood pressure, diastolic blood pressure, fasting blood sugar, smoking, total cholesterol, and body mass index. For the MrOS Study: data on diastolic blood pressure was not available. For the EPIC-Norfolk study, data on fasting blood sugar at baseline was not available, the model was adjusted for HbA1c at baseline. For the Busselton study: data on fasting blood sugar at baseline was not available, the model was adjusted for oral glucose tolerance test result at baseline;

† Incident diabetes analyzed as a risk ratio and data on number of events per category was not available for the study by Gronich et al.;

‡ value is HR (95% CI)

**Table 3:** Sensitivity analysis on subclinical hypothyroidism and incident diabetes at last available follow-up

Sensitivity analysis	No. of participants		No. of included studies	OR (95% CI)
	Euthyroid	Shypo		
1) Excluding participants with thyroid medication^*^	22215	1777	11	0.99 (0.82 - 1.21)
2) Excluding participants with thyroid-altering medication^**^	15826	1145	6	0.96 (0.77 - 1.21)
**3) Limiting analyses to participants with repeated TFT at FU**	16078	1333	6	0.96 (0.69 - 1.33)
**4) Using additional data to define diabetes** ^†^	51580	4072	17	1.05 (0.92-1.21)
**5) Limiting analyses to studies with <20% missing data at follow-up**	14073	1180	3	0.94 (0.75-1.18)
**6) Limiting analyses to high-quality studies**‡	37577	3013	17	1.02 (0.88 - 1.17)

No. of participants: total number of participants at last available follow-up; Shypo: subclinical hypothyroidism; OR: odds ratio; CI: confidence interval; TFT: thyroid function test; FU: follow-up

* Thyroid medication was defined as levothyroxine or anti-thyroid medication use;

** Thyroid-altering medication was defined as levothyroxine, anti-thyroid medication, lithium, or amiodarone use;

† If available, selfreported diabetes and linkage to medical records was used to define diabetes;

All studies were classified as good quality according to the Newcastle-Ottawa quality assessment scale for cohort studies

**Table 4:** Sensitivity analysis on subclinical hyperthyroidism and incident diabetes at last available follow-up

Sensitivity analysis	No. of participants		No. of included studies	OR (95% CI)
	Euthyroid	Shyper		
1) Excluding participants with thyroid medication^*^	19765	470	9	0.91 (0.65-1.26)
2) Excluding participants with thyroid-altering medication^**^	14080	230	5	0.83 (0.49-1.40)
**3) Limiting analyses to participants with repeated TFT at FU**	13979	567	2	0.97 (0.36-2.60)
**4) Using additional data to define diabetes** ^†^	44928	1584	12	1.00 (0.81-1.22)
**5) Excluding participants without or with abnormal FT3 measurement**	15397	467	6	0.94 (0.66-1.34)
**6) Limiting analyses to studies with <20% missing data at follow-up**	13387	257	2	1.01 (0.63-1.62)
**7) Limiting analyses to high-quality studies** ^‡^	31659	1088	12	1.03 (0.82-1.30)

Shyper: subclinical hyperthyroidism; OR: odds ratio; CI: confidence interval; TFT: thyroid function test; FU: follow-up

* Thyroid medication was defined as levothyroxine or anti-thyroid medication use;

** Thyroid-altering medication was defined as levothyroxine, anti-thyroid medication, lithium, or amiodarone use;

† If available, self-reported diabetes and linkage to medical records was used to define diabetes;

‡ All studies were classified as good quality according to the Newcastle-Ottawa quality assessment scale for cohort studies

## Data Availability

Individual participant data are not publicly available due to confidentiality issues.

## Significance statement

Evidence is conflicting regarding whether an association exists between subclinical thyroid dysfunction and incident diabetes. We therefore aimed to investigate whether individuals with subclinical thyroid dysfunction are more prone to develop diabetes in the long run as compared to euthyroid individuals. We included data from 18 international cohort studies with 61,178 adults and a mean follow-up time of 8.2 years. We did not find an association between subclinical hypothyroidism or subclinical hyperthyroidism at baseline and incident diabetes at follow-up. Our results have clinical implications as they do not support screening patients with subclinical thyroid dysfunction for diabetes nor treating them in the hope of preventing diabetes in the future.

## References

[1] Biondi B, Kahaly GJ, Robertson RP (2019). Thyroid Dysfunction and Diabetes Mellitus: Two Closely Associated Disorders. Endocr Rev.

[2] Biondi B, Cooper DS (2008). The clinical significance of subclinical thyroid dysfunction. Endocr Rev.

[3] Rodondi N, den Elzen WP, Bauer DC, Cappola AR, Razvi S, Walsh JP, Asvold BO, Iervasi G, Imaizumi M, Collet TH (2010). Subclinical hypothyroidism and the risk of coronary heart disease and mortality. JAMA.

[4] Collet TH, Gussekloo J, Bauer DC, den Elzen WP, Cappola AR, Balmer P, Iervasi G, Asvold BO, Sgarbi JA, Volzke H (2012). Subclinical hyperthyroidism and the risk of coronary heart disease and mortality. Arch Intern Med.

[5] Fleiner HF, Bjoro T, Midthjell K, Grill V, Asvold BO (2016). Prevalence of Thyroid Dysfunction in Autoimmune and Type 2 Diabetes: The Population-Based HUNT Study in Norway. J Clin Endocrinol Metab.

[6] Bensenor IM, Goulart AC, Molina Mdel C, de Miranda EJ, Santos IS, Lotufo PA (2015). Thyrotropin Levels, Insulin Resistance, and Metabolic Syndrome: A Cross-Sectional Analysis in the Brazilian Longitudinal Study of Adult Health (ELSA-Brasil). Metab Syndr Relat Disord.

[7] Roos A, Bakker SJ, Links TP, Gans RO, Wolffenbuttel BH (2007). Thyroid function is associated with components of the metabolic syndrome in euthyroid subjects. J Clin Endocrinol Metab.

[8] Hage M, Zantout MS, Azar ST (2011). Thyroid disorders and diabetes mellitus. J Thyroid Res.

[9] Rezzonico J, Rezzonico M, Pusiol E, Pitoia F, Niepomniszcze H (2008). Introducing the thyroid gland as another victim of the insulin resistance syndrome. Thyroid.

[10] Chaker L, Ligthart S, Korevaar TI, Hofman A, Franco OH, Peeters RP, Dehghan A (2016). Thyroid function and risk of type 2 diabetes: a population-based prospective cohort study. BMC Med.

[11] Mehran L, Amouzegar A, Bakhtiyari M, Mansournia MA, Rahimabad PK, Tohidi M, Azizi F (2017). Variations in Serum Free Thyroxine Concentration Within the Reference Range Predicts the Incidence of Metabolic Syndrome in Non-Obese Adults: A Cohort Study. Thyroid.

[12] Waring AC, Rodondi N, Harrison S, Kanaya AM, Simonsick EM, Miljkovic I, Satterfield S, Newman AB, Bauer DC, Health A (2012). Thyroid function and prevalent and incident metabolic syndrome in older adults: the Health, Ageing and Body Composition Study. Clin Endocrinol (Oxf).

[13] Rong F, Dai H, Wu Y, Li J, Liu G, Chen H, Zhang X (2021). Association between thyroid dysfunction and type 2 diabetes: a meta-analysis of prospective observational studies. BMC Med.

[14] Tierney JF, Vale C, Riley R, Smith CT, Stewart L, Clarke M, Rovers M (2015). Individual Participant Data (IPD) Meta-analyses of Randomised Controlled Trials: Guidance on Their Use. PLoS Med.

[15] Stewart LA, Clarke M, Rovers M, Riley RD, Simmonds M, Stewart G, Tierney JF (2015). Group P-ID.Preferred Reporting Items for Systematic Review and Meta-Analyses of individual participant data: the PRISMA-IPD Statement. JAMA.

[16] Wells GSB, O’Connell D, Peterson J, Welch V, Losos M, Tugwell P (2016). The Newcastle-Ottawa Scale (NOS) for assessing the quality of nonrandomised studies in meta-analyses.

[17] Atkins D, Best D, Briss PA, Eccles M, Falck-Ytter Y, Flottorp S, Guyatt GH, Harbour RT, Haugh MC, Henry D (2004). Grading quality of evidence and strength of recommendations. BMJ.

[18] Blum MR, Bauer DC, Collet TH, Fink HA, Cappola AR, da Costa BR, Wirth CD, Peeters RP, Asvold BO, den Elzen WP (2015). Subclinical thyroid dysfunction and fracture risk: a meta-analysis. JAMA.

[19] Hollowell JG, Staehling NW, Flanders WD, Hannon WH, Gunter EW, Spencer CA, Braverman LE, Serum TSH (2002). T(4), and thyroid antibodies in the United States population (1988 to 1994): National Health and Nutrition Examination Survey (NHANES III). J Clin Endocrinol Metab.

[20] Schneider C, Feller M, Bauer DC, Collet TH, da Costa BR, Auer R, Peeters RP, Brown SJ, Bremner AP, O’Leary PC (2018). Initial evaluation of thyroid dysfunction - Are simultaneous TSH and fT4 tests necessary. PLoS One.

[21] American Diabetes A (2021). 2. Classification and Diagnosis of Diabetes: Standards of Medical Care in Diabetes-2021. Diabetes Care.

[22] Gronich N, Deftereos SN, Lavi I, Persidis AS, Abernethy DR, Rennert G (2015). Hypothyroidism is a Risk Factor for New-Onset Diabetes: A Cohort Study. Diabetes Care.

[23] Boekholdt SM, Titan SM, Wiersinga WM, Chatterjee K, Basart DC, Luben R, Wareham NJ, Khaw KT (2010). Initial thyroid status and cardiovascular risk factors: the EPIC-Norfolk prospective population study. Clin Endocrinol (Oxf).

[24] Ceresini G, Ceda GP, Lauretani F, Maggio M, Usberti E, Marina M, Bandinelli S, Guralnik JM, Valenti G, Ferrucci L (2013). Thyroid status and 6-year mortality in elderly people living in a mildly iodine-deficient area: the aging in the Chianti Area Study. J Am Geriatr Soc.

[25] Gussekloo J, van Exel E, de Craen AJ, Meinders AE, Frolich M, Westendorp RG (2004). Thyroid status, disability and cognitive function, and survival in old age. JAMA.

[26] Nanchen D, Gussekloo J, Westendorp RG, Stott DJ, Jukema JW, Trompet S, Ford I, Welsh P, Sattar N, Macfarlane PW (2012). Subclinical thyroid dysfunction and the risk of heart failure in older persons at high cardiovascular risk. J Clin Endocrinol Metab.

[27] Walsh JP, Bremner AP, Bulsara MK, O’Leary P, Leedman PJ, Feddema P, Michelangeli V (2005). Subclinical thyroid dysfunction as a risk factor for cardiovascular disease. Arch Intern Med.

[28] Norman PE, Flicker L, Almeida OP, Hankey GJ, Hyde Z, Jamrozik K (2009). Cohort Profile: The Health In Men Study (HIMS). Int J Epidemiol.

[29] Rodondi N, Bauer DC, Cappola AR, Cornuz J, Robbins J, Fried LP, Ladenson PW, Vittinghoff E, Gottdiener JS, Newman AB (2008). Subclinical thyroid dysfunction, cardiac function, and the risk of heart failure. The Cardiovascular Health study. J Am Coll Cardiol.

[30] Rogers WJ, Alderman EL, Chaitman BR, Di Sciascio G, Horan M, Lytle B, Mock MB, Rosen AD, Sutton-Tyrrell K, Weiner BH (1995). Bypass Angioplasty Revascularization Investigation (BARI): baseline clinical and angiographic data. Am J Cardiol.

[31] Smink PA, Lambers Heerspink HJ, Gansevoort RT, de Jong PE, Hillege HL, Bakker SJ, de Zeeuw D (2012). Albuminuria, estimated GFR, traditional risk factors, and incident cardiovascular disease: the PREVEND (Prevention of Renal and Vascular Endstage Disease) study. Am J Kidney Dis.

[32] Vanderpump MP, Tunbridge WM, French JM, Appleton D, Bates D, Clark F, Evans Grimley J, Hasan DM, Rodgers H, Tunbridge F (1995). The incidence of thyroid disorders in the community: a twenty-year follow-up of the Whickham Survey. Clin Endocrinol (Oxf).

[33] Waring AC, Harrison S, Samuels MH, Ensrud KE, Le BES, Hoffman AR, Orwoll E, Fink HA, Barrett-Connor E, Bauer DC (2012). Thyroid function and mortality in older men: a prospective study. J Clin Endocrinol Metab.

[34] Moon JH, Park YJ, Kim TH, Han JW, Choi SH, Lim S, Park DJ, Kim KW, Jang HC (2014). Lower-but-normal serum TSH level is associated with the development or progression of cognitive impairment in elderly: Korean Longitudinal Study on Health and Aging (KLoSHA). J Clin Endocrinol Metab.

[35] Rojo-Martinez G, Valdes S, Soriguer F, Vendrell J, Urrutia I, Perez V, Ortega E, Ocon P, Montanya E, Menendez E (2020). Incidence of diabetes mellitus in Spain as results of the nation-wide cohort di@bet.es study. Sci Rep.

[36] Soriguer F, Rojo-Martinez G, Almaraz MC, Esteva I, Ruiz de Adana MS, Morcillo S, Valdes S, Garcia-Fuentes E, Garcia-Escobar E, Cardona I (2008). Incidence of type 2 diabetes in southern Spain (Pizarra Study). Eur J Clin Invest.

[37] Chang CH, Yeh YC, Shih SR, Lin JW, Chuang LM, Caffrey JL, Tu YK (2017). Association between thyroid dysfunction and dysglycaemia: a prospective cohort study. Diabet Med.

[38] Han C, He X, Xia X, Li Y, Shi X, Shan Z, Teng W (2015). Subclinical Hypothyroidism and Type 2 Diabetes:A Systematic Review and Meta-Analysis. PLoS One.

[39] Chubb SA, Davis WA, Inman Z, Davis TM (2005). Prevalence and progression of subclinical hypothyroidism in women with type 2 diabetes: the Fremantle Diabetes Study. Clin Endocrinol (Oxf).

