# Supplementary material for: Subclinical thyroid dysfunction and incident diabetes: a systematic review and an individual participant data analysis of prospective cohort studies
Source: Eur J Endocrinol. Author manuscript; Available in PMC 2022 Nov 22. (PMC7613845; doi:10.1530/EJE-22-0523)
Supplement: Supplementary Material [file EMS156769-supplement-Supplementary_Material.pdf]

Supplementary Figure 1. Flow diagram of the systematic review and study selection

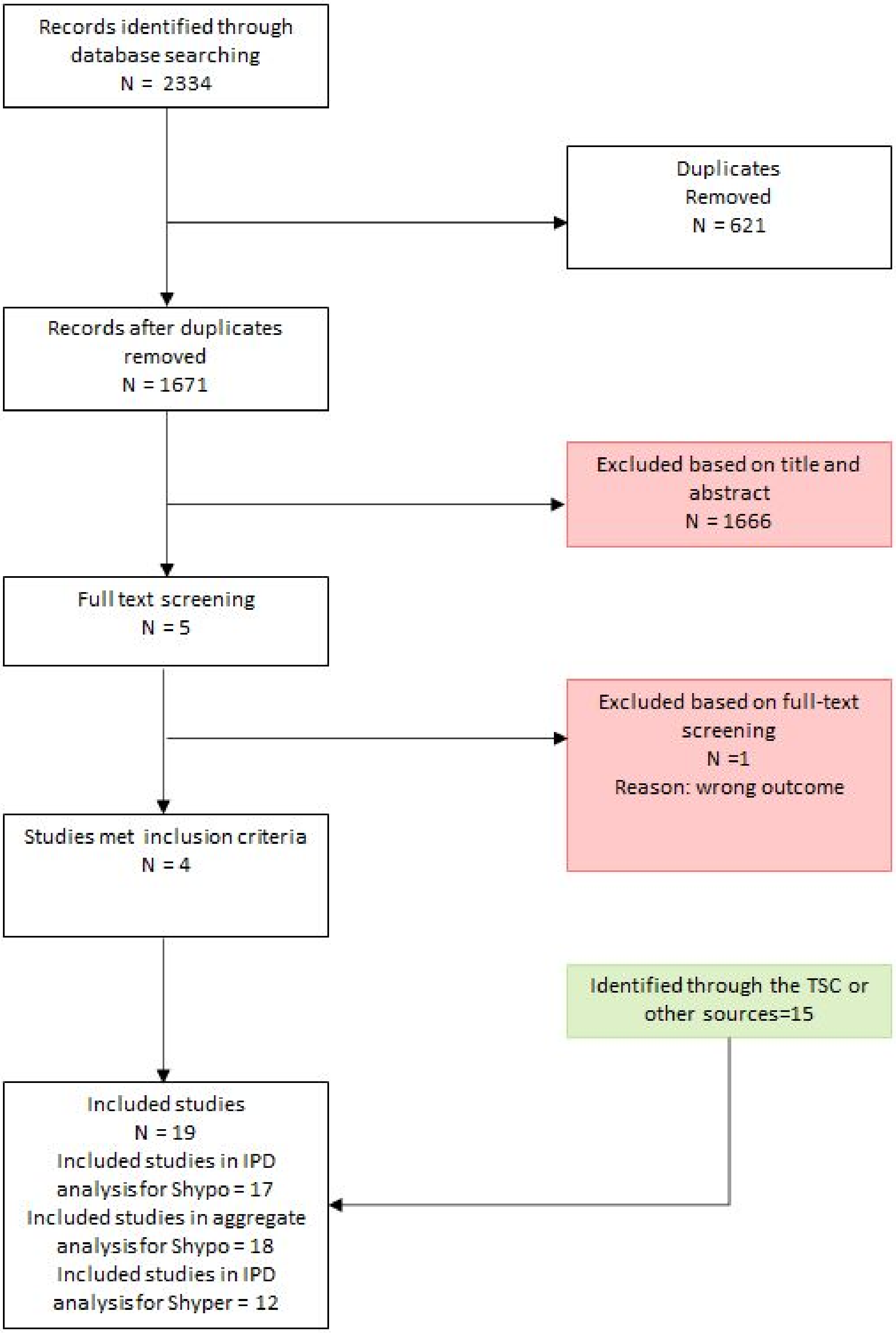

Abbreviations: IPD: individual participant data; Shypo: Subclinical hypothyroidism; Shyper: subclinical hyperthyroidism; TSC: Thyroid Studies Collaboration

Supplementary Table 1. Institutional review board information for cohort studies

Study	Institutional review board information
PROSPER Study	Approved by the institutional ethics review boards of centers of Cork University (Ireland), Glasgow University (Scotland) and Leiden University Medical Center (the Netherlands)
ELSA-Brasil	CAAE 0017.1.069.000-06, 0186.1.203.000-06, 08 1096 12.7.2003 5060, 0058.0.011.000-07, 0016.1.198.000-06, 0017.1.069.000-06, 0017.1.069.000-06, 0186.1.203.000-06, 08 1096 12.7.2003 5060, 0058.0.011.000-07, 0016.1.198.000-06, 0058.0.011.000-07
BARI Study	n.847/DG/2011
Leiden-85+ Study	P97/04
KLoSHA Study	B-0912-089-010, B-0912-089-005, B-2006-618-103
Rotterdam Study	MEC 02.1015, 1071272-159521-PG.
Pizarra Study	Provincial Research Ethics Committee of Malaga 21-February 2019
MrOS	20200148, 10-04683
Di@bet.es Study	Provincial Research Ethics Committee of Malaga 27-October 2016
PREVEND Study	MEC/96/01/022d
HIMS	RA/4/1/5765
InChianti Study	656
Health ABC Study	11-AG-N079
EPIC-Norfolk Study	REC Ref: 98CN01
Tehran Thyroid Study	EC89/05/13
CHS	843156
Busselton Health Study	RA 03/056, RA/4/1/2077
Whickham Survey	Institutional Review Board number not available. All participants gave informed consent.

PROSPER: prospective study of Pravastatin in the elderly at risk Study; ELSA-Brasil: Brazilian Longitudinal Study of Adult Health; Leiden 85+ Study: Leiden 85-plus Study; KLoSHA: Korean Longitudinal Study on Health and Aging Study; MrOS: Osteoporotic Fractures in Men Study; PREVEND: Prevention of Renal and Vascular End-stage Disease Study; HIMS: Health in Men Study; InChianti: Invecchiare in Chianti Study; Health ABC Study: The Health, Aging and Body Composition Study; EPIC-Norfolk Study: European Prospective Investigation into Cancer - Norfolk Study; CHS: Cardiovascular Health Study

Supplementary Table 2. Quality assessment using the Newcastle-Ottawa quality assessment scale for cohort studies

The first category refers to the representativeness of the study. The criterion was met if conducted in the general population, including both sexes, and adults in each age. The second category refers to selection of the non-exposed. Criterion was met if studies had euthyroid controls. The third category refers to ascertainment of exposure. The criterion was met if studies used an appropriate TSH measurement. The fourth category refers to demonstration that outcome of interest was not present at start of the study. Criterion was met if individuals with diabetes were excluded from analyses. The fifth category refers to comparability on the basis of the design or analysis. Criterion was met when analyses were adjusted for at least age. The sixth category refers to assessment of the outcome, criterion was met if data on diabetes status at follow-up was available. The seventh category refers to the length of follow-up. Criterion was met if the follow-up was at least one year. The category eight refers on the adequacy of follow-up, criterion was met if missing data at follow-up ≤ 20%. We considered the final NOS quality score of studies for judging the study limitations (risk of bias) in the GRADE assessment

Study	Selection				Comparability	Outcome			
	**Representativeness of exposed cohort**	**Selection of the non-exposed cohort**	**Ascertainment of exposure**	**Demonstration that outcome of interest was not present at start of the study**	**Comparability of cohorts on the basis of the design or analysis***	**Assessment of outcome**	**Was follow-up long enough for outcomes to occur**	**Adequacy of follow up (Missing data on primary outcome at follow-up)***	**Quality Score**
PROSPER Study	-	★	★	★	★★	★	★	★	8 Good quality
ELSA-Brasil Study	★	★	★	★	★★	★	★	★	9 Good quality
BARI Study	-	★	★	★	★★	★	★	★	8 Good quality
Leiden 85+ Study	★	★	★	★	★★	★	★	-	7 Good quality
KLoSHA Study	★	★	-	★	★★	★	★	-	7 Good quality
Rotterdam Study	★	★	★	★	★★	★	★	-	8 Good quality
Pizarra Study	★	★	★	★	★★	★	★	-	8 Good quality
MrOS	★	★	★	★	★	★	★	-	7 Good quality

Di@bet.es Study	★	★	★	★	★★	★	★	-	8<br>Good quality
PREVEND Study	★	★	★	★	★★	★	★	-	8<br>Good quality
HIMS	★	★	★	★	★	★	★	★	8<br>Good quality
InChianti Study	★	★	★	★	★★	★	★		8<br>Good quality
Health ABC Study	★	★	★	★	★★	★	★	-	8<br>Good quality
EPIC-Norfolk Study	★	★	★	★	★★	★	★	-	8<br>Good quality
Tehran Thyroid Study	★	★	★	★	★★	★	★	-	8<br>Good quality
CHS	★	★	★	★	★★	★	★	-	8<br>Good quality
Busselton Health Study	★	★	★	★	★★	★	★	-	8<br>Good quality
Whickham Survey	★	★	-	★	★★	★	★	-	7<br>Good quality
Gronich et al[22]	★	★	★	★	★★	★	-	-	7<br>Good quality

*Primary outcome adjusted for age and sex

Thresholds for converting the Newcastle-Ottawa scales to AHRQ standards (good, fair, and poor): Good quality: 3 or 4 stars in selection domain AND 1 or 2 stars in comparability domain AND 2 or 3 stars in outcome/exposure domain Fair quality: 2 stars in selection domain AND 1 or 2 stars in comparability domain AND 2 or 3 stars in outcome/exposure domain Poor quality: 0 or 1 star in selection domain OR 0 stars in comparability domain OR 0 or 1 stars in outcome/exposure domain.

Abbreviations: PROSPER: prospective study of Pravastatin in the elderly at risk; ELSA-Brasil: Brazilian Longitudinal Study of Adult Health; Leiden 85 + Study: Leiden 85 plus Study; KLoSHA: Korean Longitudinal Study on Health and Aging; MrOS: Osteoporotic Fractures in Men Study; PREVEND: Prevention of Renal and Vascular End-stage Disease; HIMS: Health in Men Study; InChianti Study: Invecchiare in Chianti Study; Health ABC Study: The Health, Aging and Body Composition study; EPIC-Norfolk Study: European Prospective Investigation into Cancer - Norfolk study; CHS: Cardiovascular Health Study.

Supplementary Table 3. Grading of Recommendation Assessment, Development and Evaluation (GRADE)

Question: Participants with subclinical Hypo- or hyperthyroidism compared to euthyroid controls for incident diabetes during follow-up

Certainty assessment							Number of patients		Effect	Certainty
**№ of studies**	**Study design**	**Risk of bias/ Study Limitations**	**Inconsistency**	**Indirectness**	**Imprecision**	**Other considerations**	**Exposed Group**	**Control Group**	**Absolute (95% CI)**	
Incident diabetes **(SHypo) (dichotomous outcome: yes/no)**										
17	observational studies	not serious [a]	not serious	not serious	not serious	none	225/3064	2656/38691	**1 more per 1000** (from 8 fewer to 12 more)	⨁⨁◯◯ LOW *
Incident diabetes **(SHyper (dichotomous outcome: yes/no)**										
12	observational studies	not serious	not serious	not serious	not serious	none	89/1128	2301/31644	3 more per 1000 (from 13 fewer to 22 more)	⨁⨁◯◯ LOW *

Abbreviations: CI: Confidence interval; Explanations: **Certainty: High,** Further research is very unlikely to change our confidence int the estimate of effect, **Moderate:** Further research is likely to have an important impact on our confidence in the estimate of effect and may change the estimate. **Low:** Further research is very likely to have an important impact on our confidence in the estimate of effect and is likely to change the estimate, **Very low:** Any estimate of effect is very uncertain[14]

*Certainty was low because of downgrading due to the observational nature of the studies, according to GRADE guidelines

Supplementary Figure 2. Funnel plot for the association between subclinical hypothyroidism and incident diabetes at last available follow-up

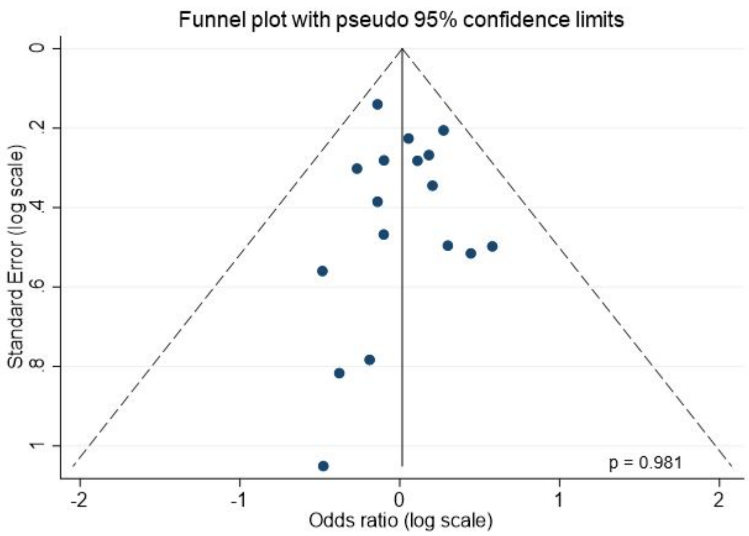

Supplementary Figure 3. Funnel plot for the association between subclinical hyperthyroidism and incident diabetes at last available follow-up

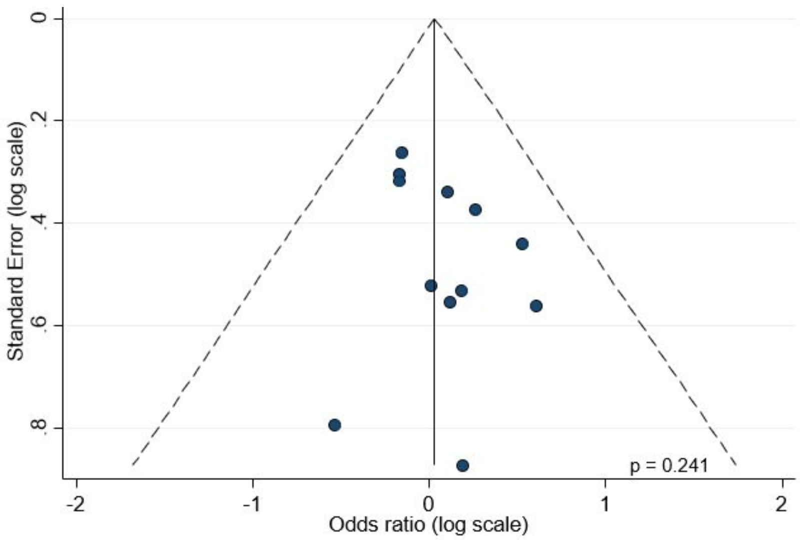

Appendix. Search strategy for Ovid Medline, Ovid Embase, and in the Cochrane Library (February 11, 2022)

Ovid Medline

((((Thyroid Disease/ or Hyperthyroidism/ or Hypothyroidism/ or Thyroid Hormones/ or Triiodothyronine/ or Thyroid Function Tests/ or Thyroxine/ or exp Thyrotropin/ and (subclinical or sub-clinical or mild or latent or subnormal or pre-clinical or preclinical).ab,ti,kw.) or (((subclinical or sub-clinical or mild or latent or subnormal or pre-clinical or preclinical) adj6 (hypothyroid* or hyperthyroid* or thyroid dysfunction* or triodothyronin* or triiodothyronin* or thyroxin* or liothyronin* or TSH or T4 or ft4 or t 4 or ft 4 or T3 or thyroid failur* or thyroid diseas* or euthyroid* or graves or goiter)) or ((thyroid function or TSH) adj3 normal range)).ab,ti,kw.) and Diabetes Mellitus, Type 2/ or Metabolic Syndrome/ or exp Insulin Resistance/ or Glucose Tolerance Test/ or (((diabet* or dm) adj3 (type 2 or type2 or type ii or non insulin or noninsulin or insulin independent or adult onset or slow onset or maturity onset or ketosis resistant)) or T2DM or dmt2 or dm2 or T2-DM or dm-t2 or dm-2 or NIDDM or pre-diabet* or prediabet* or metabolic syndrome or insulin resistance).ab,ti,kw. or (Insulin/bl or Glucose Intolerance/ or Blood Glucose/ or Glucose Metabolism Disorders/ or exp Hyperinsulinism/ or Hyperglycemia/ or exp Glycated Hemoglobin A/ or (((glucose or sugar or insulin*) adj3 (level* or blood or serum or plasma* or concentration* or tolerance or intolerance or sensitivit* or insensitivit* or resistan* or response or homeosta*)) or glycemic control or glycaemic control or hyperinsulin* or hypoinsulin* or insulinaem* or insulinem* or HOMA-IR or fasting glucose or hypoglycemi* or hypoglycaemi* or hyperglycemi* or hyperglycaemi* or glycated hemoglobin or glycated haemoglobin or HbA1c or HbA-1c or Hb A1c or hemoglobin A1c or haemoglobin A1c).ab,ti,kw.))) not (exp animals/ not humans/) not (letter* or news or comment* or editorial* or congres*).pt.

Ovid Embase

((('subclinical hypothyroidism'/de OR 'subclinical hyperthyroidism'/de OR (('thyroid disease'/de OR 'hyperthyroidism'/de OR 'hypothyroidism'/de OR 'thyroid hormone'/de OR 'liothyronine'/de OR 'thyroid function'/de OR 'thyroid function test'/de OR 'thyrotropin blood level'/de OR 'thyroid hormone blood level'/exp OR 'thyroid peroxidase antibody'/exp OR 'thyroglobulin antibody'/de OR 'thyroxine'/de OR 'thyrotropin'/de) AND ((subclinical OR sub-clinical OR mild OR latent OR subnormal OR pre-clinical OR preclinical):ab,ti,kw)) OR (((subclinical OR sub-clinical OR mild OR latent OR subnormal OR pre-clinical or preclinical) NEAR/6 (hypothyroid* OR hyperthyroid* OR 'thyroid dysfunction*' OR tri*odothyronin* OR thyroxin* OR liothyronin* OR TSH OR T4 OR ft4 OR 't 4' OR 'ft 4' OR T3 OR 'thyroid failur*' OR 'thyroid diseas*' OR euthyroid* OR graves OR goiter)) OR (('thyroid function' OR TSH) NEAR/3 'normal range')):ab,ti,kw)) AND (('non insulin dependent diabetes mellitus'/de OR 'metabolic syndrome X'/de OR 'insulin resistance'/de OR 'oral glucose tolerance test'/de OR (((diabet* OR dm) NEAR/3 ('type 2' OR type2 OR 'type ii' OR 'non insulin' OR noninsulin OR 'insulin independent' OR 'adult onset' OR 'slow onset' OR 'maturity onset' OR 'ketosis resistant')) OR T2DM OR dmt2 OR dm2 OR T2-DM OR dm-t2 OR dm-2 OR NIDDM OR pre-diabet* OR prediabet* OR 'metabolic syndrome' OR 'insulin resistance'):ab,ti,kw) OR ('insulin response'/de OR 'impaired glucose tolerance'/de OR 'glucose blood level'/exp OR 'glycemic control'/de OR 'insulin blood level'/exp OR hyperinsulinism/exp OR 'homa ir'/de OR 'hyperglycemia'/de OR 'hemoglobin A1c'/de OR 'glycosylated hemoglobin'/exp OR 'fasting glucose'/de OR (((glucose OR sugar OR insulin*) NEAR/3 (level* OR blood OR serum OR plasma* OR concentration* OR tolerance OR intolerance OR sensitivit* OR insensitivit* OR resistan* OR response OR homeosta*)) OR 'glycemic control' OR 'glycaemic control' OR hyperinsulin* OR hypoinsulin* OR insulinaem* OR insulinem* OR HOMA-IR OR 'fasting glucose' OR hypoglycemi* OR hypoglycaemi* OR hyperglycemi* OR hyperglycaemi* OR 'glycated hemoglobin' OR 'glycated haemoglobin' OR HbA1c OR HbA-1c OR 'Hb A1c' OR 'hemoglobin A1c' OR 'haemoglobin A1c'):ab,ti,kw))) NOT ([animals]/lim NOT [humans]/lim) NOT ([Conference Abstract]/lim OR [Letter]/lim OR [Note]/lim OR [Editorial]/lim)

Cochrane Central Register of Controlled Trials

((((subclinical OR sub-clinical OR mild OR latent OR subnormal OR pre-clinical or preclinical) NEAR/6 (hypothyroid* OR hyperthyroid* OR thyroid NEXT dysfunction* OR triodothyronin* OR triiodothyronin* OR

thyroxin* OR liothyronin* OR TSH OR T4 OR ft4 OR "t 4" OR "ft 4" OR T3 OR thyroid NEXT failur* OR thyroid NEXT diseas* OR euthyroid* OR graves OR goiter)) OR (("thyroid function" OR TSH) NEAR/3 "normal range")):ab,ti,kw) AND ((((diabet* OR dm) NEAR/3 ("type 2" OR type2 OR "type ii" OR "non insulin" OR noninsulin OR "insulin independent" OR "adult onset" OR "slow onset" OR "maturity onset" OR "ketosis resistant")) OR T2DM OR dmt2 OR dm2 OR "T2 DM" OR "dm t2" OR "dm 2" OR NIDDM OR pre-diabet* OR prediabet* OR "metabolic syndrome" OR "insulin resistance"):ab,ti,kw OR (((glucose OR sugar OR insulin*) NEAR/3 (level* OR blood OR serum OR plasma* OR concentration* OR tolerance OR intolerance OR sensitivit* OR insensitivit* OR resistan* OR response OR homeosta*)) OR "glycemic control" OR "glycaemic control" OR hyperinsulin* OR hypoinsulin* OR insulinaem* OR insulinem* OR HOMA-IR OR "fasting glucose" OR hypoglycemi* OR hypoglycaemi* OR hyperglycemi* OR hyperglycaemi* OR "glycated hemoglobin" OR "glycated haemoglobin" OR HbA1c OR HbA-1c OR "Hb A1c" OR "hemoglobin A1c" OR "haemoglobin A1c"):ab,ti,kw)
